# Experimental Strategies of Mesenchymal Stem Cell Propagation: Adverse Events and Potential Risk of Functional Changes

**DOI:** 10.1155/2019/7012692

**Published:** 2019-03-06

**Authors:** Katarzyna Drela, Luiza Stanaszek, Adam Nowakowski, Zuzanna Kuczynska, Barbara Lukomska

**Affiliations:** NeuroRepair Department, Mossakowski Medical Research Centre, Polish Academy of Sciences, Warsaw, Poland

## Abstract

Mesenchymal stem cells (MSCs) are attractive candidates for cell-based tissue repair approaches. Hundreds of clinical trials using MSCs have been completed and many others are still being investigated. For most therapeutic applications, MSC propagation *in vitro* is often required. However, ex vivo culture condition is not fully physiological and may affect biological properties of MSCs including their regenerative potential. Moreover, both cell cryopreservation and labelling procedure prior to infusion may have the negative impact on their expected effect *in vivo*. The incidence of MSC transformation during *in vitro* culture should be also taken into consideration before using cells in stem cell therapy. In our review, we focused on different aspects of MSC propagation that might influence their regenerative properties of MSC. We also discussed the influence of different factors that might abolish MSC proliferation and differentiation as well as potential impact of stem cell senescence and aging. Despite of many positive therapeutic effects of MSC therapy, one has to be conscious about potential cell changes that could appear during manufacturing of MSCs.

## 1. Introduction

**Figure 1:**
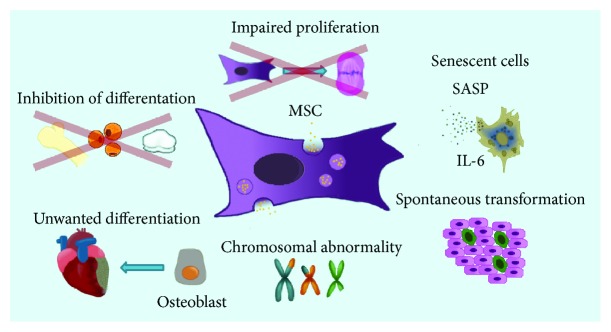
Scheme of potential risks of adverse events during MSC *in vitro* culture.

Mesenchymal stem cells (MSCs) are one of the most exploited cell types in regenerative medicine of many clinical applications. Most of scientific reports demonstrate the positive aspects of cell therapy not quoting the adverse events that might influence the quality of the final product. However, it is important to bear in mind that MSC is the heterogeneous cell population with different regenerative properties that may affect the final result of stem cell treatment. Moreover, the crucial fact is the source of MSC isolation and the age of the donor. It is already known that the population of MSCs obtained from older or diseased patients is different in comparison to young and healthy people. Due to the infrequent presence of MSCs within tissues, *in vitro* propagation is almost always required to achieve a sufficient cell number for *in vivo* application. Unfortunately, *in vitro* cell multiplication is not neutral for MSC biological properties. In our review, the potential risks of MSC culture are highlighted including the effects of multiple, frequently nonphysiological conditions during *in vitro* culture such as oxygen level, cell density, medium content, passage number, and proliferative senescence that altogether may alter MSC condition (Figure 1). Additionally, the influences of cryopreservation methods are discussed. Furthermore, we point out the aspects of MSC labelling that should be applied with caution and consideration of its influence on the labelled cells.

## 2. Diversity of MSCs

The first reports on adult stem cells isolated from bone marrow appeared more than 50 years ago. At the beginning, the scientists proposed the term mesenchymal stem cells (MSCs), initially referred to multilineage progenitor cells showing three lineage differentiation abilities. It has been demonstrated that MSCs are heterogeneous cells with several populations significantly varying in regenerative potentials. Recent findings confirmed that these cells comprise only part of the stromal cell population [1, 2]. Subsequently, in nomenclature, the definition “mesenchymal stromal cells” was proposed for MSCs as that name would reflect the stromal origin of the cells [1]. Lately, Caplan and other researchers proposed for MSCs the term “medical signaling cells” [3]. They suggested that MSC multipotency is not a pivotal aspect of cell therapy and thus primarily referred to their paracrine function as a major activity in tissue repair [4]. It needs to be underlined that regarding diverse MSC tissue origin and/or donors, secretome of the cells might differ [5]. Moreover, recent research demonstrated that MSCs isolated from diseased patients might release factors that might negatively influence regenerative processes [6]. Furthermore, there are some controversies regarding MSC ontogeny. Some researchers suggest that MSCs are present in almost all tissues, being located in perivascular niches, which suggests their close relation to pericytes [7, 8]. Such controversial view on MSC origin was demonstrated by Takashima and colleagues suggesting that the first population of MSCs during development is derived from the neuroectodermal neural crest cells [9]. Further investigation by Coste's group proved that the bone marrow contains population of cells with neural crest features which are able to differentiate into neurons and Schwann cells [10]. The neuroectodermal origin could explain the neural differentiation potential of MSCs documented in many recent papers [11].

Clinical applications of MSCs have been tested for many years. So far, bone marrow-derived MSCs are the most often used type of cells in more than 50% of clinical trials [12]. Since the aspiration of bone marrow tissue is a traumatic and painful procedure, the researchers quest for an alternative source for MSCs. Recently, MSC population have been isolated from the tissues that are discarded such as umbilical cord, placenta, or fat tissue obtained from liposuction procedures. It has been shown that neonatal tissues have biological advantages in comparison to adult sources that make them a useful source for stem cells including MSCs. Neonatal tissues are easily available, which avoid invasive technical procedures and they are ethically noncontroversial. It was shown that MSCs derived from neonatal tissues have higher biological capacities than MSCs obtained from adult sources. Several studies showed better proliferative capacity, long lifespan, and efficient differentiation potential of MSCs from birth-associated tissues over BM-MSCs [13, 14]. Moreover, neonatal tissues may contain embryonic or premature cells including the cells at earlier stage of development [15]. Nowadays, it is still unclear, which tissue source is optimal to obtain MSCs for clinical transplantation. Unanswered question is whether MSCs obtained from the different sources are still the same cells or represent a different population. These subsets of cells can be isolated from the different niches, each with distinct potential for therapeutic applications.

## 3. Potential Risks during MSC Expansion *In Vitro* Affecting Therapeutic Properties

### 3.1. MSC Culture *In Vitro*

Although hMSCs are present in almost all tissues, their frequency is relatively low [2]. Most of clinical protocols recommend 20–100 × 10^6^ hMSCs per treatment; therefore, in order to obtain such a high number of cells, hMSCs have to be expanded *in vitro* for at least 4–8 weeks before transplantation. To achieve positive results in clinical application, it is important to get high number of therapeutic cells. MSC isolation protocols rely on the ability of these cells to adhere to the plastic surface; however, this method leads to a heterogeneous population of stem cells with a substantial population of progenitor cells and also other cell types [16]. The other method is the isolation of MSCs using mechanical explant approach or proteolytic enzyme treatment with collagenase, which may impact on biological properties of the isolated cells [17–19]. Moreover, the expansion of MSCs using traditional plastic adherence cell culture in media supplemented with serum and continuous exposure to oxygen could result in the decrease of proliferative and differentiation potential with each subsequent passage. It is recently reported that oxygen level is a crucial factor in stem cell biology. Physiologically, MSCs are settled in hypoxic niches where oxygen concentration ranges between 2 and 7% [20]. Therefore, in many recent studies, hypoxic culture condition is tested in terms of MSC functions. In most experiments, MSC proliferation rate was increased in hypoxic conditions (5%) compared to standard atmospheric (21%) oxygen level culture [21]. Moreover, *in vitro* culture of hMSCs in low concentration of oxygen significantly increased cell lifespan, limited oxidative stress, DNA damage, telomere shortening, and chromosomal aberrations. At the same time, MSCs expanded in 5% oxygen level presented HIF-1*α* expression leading to improvement of several MSC functions influencing on their therapeutic potential while maintaining stable karyotype. In addition, hypoxia preconditioning was reported to increase the angiogenetic properties of MSCs [22–25]. Unfortunately, the expansion of human stem cells *in vitro* before their transplantation is usually performed at 20% O_2_. The results obtained by Estrada and coworkers suggest that cell culture at low oxygen tension favoured the metabolic state of MSCs, increased glycolysis, and diminished oxidative phosphorylation [26]. Culturing MSCs at 20% O_2_ changed their metabolism and significantly increased level of oxidative stress. Moreover, the group of Vertelov and colleagues demonstrated that hypoxic conditions influenced on MSC motility toward the injury site [27]. Recent data underlines the importance of culturing of MSCs in hypoxic condition that might increase regenerative potential of the cells such as self-renewing capacity and immunomodulatory properties [28]. Therefore, it is crucial that stem cells designated for human application should be expanded in low oxygen condition to augment their biologic potential.

Even though transplantation of MSCs has emerged as an effective method to treat diseased or damaged tissues, the most of MSCs used for clinical trials are prepared in different laboratories with often insufficient manufacturing quality control. In particular, many laboratories do not have standardized procedures for *in vitro* isolation of MSCs from different tissue samples, resulting in heterogeneous population of cells, which lead to unrepeatable experimental and clinical results. Criteria defined by ISCT guidelines for MSC characterization are not enough to identify MSCs and could be addressed for many other cell types [29]. This fact causes a lot of discrepancies in the establishment of the real nature of MSCs and their impact on tissue repair.

Another aspect that should be considered is the influence of culture reagents, i.e., proteolytic enzymes to detach cells, culture media, and storage reagents on biological properties of expanded MSCs [30]. The addition of fetal bovine serum commonly used in culture media is questionable, since the components and growth factors present in the serum are not well characterized. The ideal culture media for MSC expansion ex vivo should contain standard defined nutrients accordingly to the cell type. Moreover, proteolytic enzymes used for cell passaging or tissue digestion could promote extracellular matrix (ECM) protein damage and thus may modify activation of variety of intracellular signaling pathways [31]. In most cases, expanded MSCs have to be stored frozen until the time of treatment and the storage condition could alter the quality of the product. Some preclinical reports demonstrating that cryopreservation does not affect biological properties of MSCs, i.e., marker expression, proliferation rate, or differentiation potential [32]. However, the other studies have shown that cryopreservation could exert many adverse events and alter MSC phenotypes and cell function. The optimal freezing and thawing temperature control, cryopreservation media, and long-term storage in liquid nitrogen are the main key factors that should be considered. Recently, the new strategies and cryoprotective agents, e.g., polyvinylpyrrolidone PVP, methylcellulose, and trehalose for MSC storage, have been demonstrated [33, 34]. However, the freezing media consisting DMSO, despite of its toxicity for many types of cells, are still the most frequently used cryopreserving solution. Adverse effects of DMSO were reported both in animals and humans [35, 36]. Because of the toxic effect of DMSO, it is recommended to remove it from the thawed cells before transplantation usually using the washing steps in isotonic solutions. There are reports showing that after the infusion of MSCs previously frozen in DMSO, the patients developed transient complications including headache, vomiting, and hyper or hypotension; some of them presented respiratory and cardiovascular problems [37]. Hydroxyethyl starch (HES) commonly used for freezing different cell types could be another alternative for DMSO in cell cryoprotection [38]. Nevertheless, up to now, it was not tested in protocols for MSC cryopreservation.

### 3.2. Impaired Proliferation of MSCs

MSCs cultured *in vitro* have usually high proliferation properties; however, cell proliferation rate depends on many factors, i.e., source of MSCs, the stage of cells, or culture condition. It has been shown that neonatal MSCs have higher proliferation potential in comparison to MSCs derived from adult sources [13]. However, nonoptimal culture condition could alter MSC proliferation. Cell seeding density may have the impact on MSC growth. The initial cell number of mononuclear cells from the whole fraction of the bone marrow is a key factor to obtain efficient population of pure MSCs. Mareschi and coworkers evaluated influence of initial cell density and the time of culture on morphology, metabolic activity, and differentiation potential of BM-MSCs [39]. They have found that the optimal cell growth occurs at a lower initial culture density. MSC culture derived from initial high cell plating densities results in increased number of flat cells and low proliferation rate. However, there is a limit of low cell culture density and the samples platted at 10-100 cells/cm^2^ did not expand effectively. The recent data revealed that the best results for clinical application were obtained when the whole BM-MSC population were expanded at the density of 10.000/cm^2^ [39].

Current observations suggest that MSC expansion in three-dimensional (3D) structures could increase their therapeutic potential when compared to traditional two-dimensional (2D) culture [40]. Different culture methods have been employed to obtain efficient 3D spheroids of MSCs. Recently, an efficient method to produce MSC aggregates was proposed by Bogdanova et al. MSCs derived from adipose tissue (AT-MSCs) cultured *in vitro* in a special condition spontaneously formed aggregate-3D bodies and expressed pluripotent markers including Nanog and Oct3/4 [41]. The authors suggest that in 3D culture, MSCs retain their natural features of stem cells. However, cell aggregates can reach a big size and the injection of a pool of such condensed cells into the tissue may have detrimental effect. The biomass aggregates could end with pulmonary embolism or infarctions after systemic delivery. Moreover, not all cells in 3D bodies are at the same differentiation stage [42].

In terms of clinical transplantation, it is important to culture MSCs in media depriving of animal components, i.e., bovine products that have a potential risk of viral transmission or prion diseases as well as immune system activation of graft recipients. Autologous or allogeneic-derived plasma or platelet lysates represent more safe substitutes than FBS and allow efficient proliferation of MSCs [43, 44]. Nevertheless, some of the reports indicate the possibility of using commercially available xeno-free media for MSC culture [45, 46]. Recent reports demonstrated that the use of human platelet lysates (hPL) results in enhanced proliferation without changing morphology and function of MSCs [39]. Smith and coworkers optimized GMP expansion protocol for umbilical cord-derived MSCs (UC-MSCs) supplemented with different concentration of hPL. They observed that MSCs cultured in media enriched with 10% of platelet-rich plasma (PRP) have the most efficient proliferation rate [47]. Similar results confirming efficacy of using platelet lysates have been demonstrated in BM-MSC culture [43] However, it was shown that autologous plasma obtained from elderly patients diminished MSC proliferation properties [48, 49].

### 3.3. Unsettled Differentiation of MSCs

There are some other risk factors which should be taken into consideration before clinical transfer of MSCs. Among them, one factor is the incorrect differentiation ability of MSCs. According to ISCT minimal criteria, MSCs should differentiate into three mesodermal lineages including adipocytes, osteocytes, and chondrocytes [1]. However, the results obtained from *in vitro* studies suggested the possibility of MSC differentiation into ectodermal and endodermal cells [50]. Indeed, numerous differentiation protocols have described the ability of MSCs to turn into other cell types. Recent published data demonstrated that cell senescence associated with aging is the main factor that abolished multilineage potential of MSCs [51, 52]. Furthermore, some reports indicated the data concerning spontaneous differentiation of MSCs into unwitting cell types after transplantation. Recently, it was demonstrated that transplantation of BM-MSCs into heart in mouse experimental model of cardiomyopathy led to calcification. [53]. It has been suggested that MSC differentiation is strictly regulated by specific paracrine mediators. Osteoblastic differentiation of MSCs is stimulated by epidermal growth factor (EGF), which enhances BMP9-induced early and late osteogenic markers of MSCs cultured *in vitro*. The cross talk between BMP and EGF could be beneficial to induce osteogenesis in bone repair [54]. In contrary, bone marrow-derived MSCs treated with transforming grow factor 3*β* (TGF *β*) significantly reduced alkaline phosphate activity and decreased mineral depositions [55]. The other important factor influencing MSC differentiation is the level of oxygen. It has been proven that hypoxia could block MSC osteogenic differentiation by activating the Notch pathway [56]. In contrast, MSC exposure to atmospheric oxygen develops oxygen-stress response and inhibits cell differentiation [22]. The differentiation potential of MSCs observed in vitro depends on the presence of cell composition in bone marrow stroma. Abdallah and Kassem demonstrated that adipocytes present in the bone marrow niche could exert inhibitory effect on MSC differentiation properties. Adipocytes could secrete factors that block BMP pathway towards osteoblast lineage [57]. This may have a significant adverse effect in primary cell culture containing different cell populations. The problem of unwanted differentiation of MSCs was also confirmed in *in vivo* studies in animal models of liver injury and cardiac infarction [58]. Recent data demonstrated the role of microenvironmental factors in heterotopic ossification (HO); therefore, cell therapies in orthopaedic disorders should be considered in terms of potential risks [59]. Breitbach et al. observed the phenomenon of calcification and/or ossification in murine heart tissue when unfractionated bone marrow cells or BM-MSCs were injected into the border zone of heart infarcted area after acute myocardial insult. It suggests the osteogenic differentiation of transplanted cells [53]. The authors did not provide possible rationale for unexpected MSC misdifferentiation in heart tissue. Such phenomenon has not been reported before, but it supposed to warn the scientific community that ossification process may happen following MSC injection in other than bone tissues. The calcification has been also observed by Liao and colleagues in the site of injured abdominal aorta after BM-MSC transplantation in hyperlipidemic rats [60].

The knowledge coming from experimental cardiovascular studies indicating some possible risks of MSC application may suggest that “priming” or “predifferentiation” of bone marrow cells including MSCs may be an optimal approach prior to their transplantation, enhancing their differentiation capacity into cardiomyocytes and limiting misdifferentiation of transplanted cells in the heart niche. The problem of MSC unsettled differentiation may be dissected into two different negative aspects. On one hand, there is the problem of MSC uncontrolled differentiation into unwanted linages, and on the other, the total loss of differentiation potential at all. This problem may be caused by unspecified culture condition and due to the fact that general population of multipotent MSCs may differ depending of stem cell niche.

## 4. Aging and Senescence of MSCs and Potential Risk of Transformation

Mesenchymal stem cells, similarly to other types of cells, undergo senescence after definite number of cell passages *in vitro*. Many papers have demonstrated that long-term expansion of MSCs could induce cellular senescence that is associated with growth arrest and cell apoptosis as well as with a reduction of their “medical” properties [51]. However, the exact mechanism of MSC senescence and molecular signaling pathways still remain unknown. Ko and coworkers have shown that similarly to primary tissue cells, umbilical cord blood-derived MSCs (UCB-MSCs) initiate permanent arrest of cell division after 50 population doublings (PDs), but the process of MSC senescence depends mainly on the source of MSCs [61]. The data published by Stab's group revealed that AT-MSCs developed a senescent phenotype at seventh population doubling, characterized by significant change in cell morphology with an increase in *β*-galactosidase expression [62]. The changes associated with senescence are characterized by heterogeneity of cells expressing different phenotypic markers. Previous studies established that MSC aging correlates with the decline of their functions. This process is characterized by phenotype alteration observed in culture including changes in morphology, reduction of telomere length, and decrease in immunological properties of MSCs [63–65]. Thus, an understanding of the molecular mechanisms controlling MSC senescence is very important to determine age-associated MSC alterations. Furthermore, this knowledge is fundamental for the development of therapeutic intervention that can slow down or reverse age-related degenerative changes and improve repair processes [66]. Bellotti's group indicated that besides *β*-galactosidase, prelamin A level is a useful marker for detection of senescent MSCs. They confirmed that this nuclear protein level provided an easy and reliable tool to screen MSCs before their use in clinical application [67]. Presently, most of the researchers put special emphasis to the role of oxidative stress to elucidate the mechanism of cellular aging. Recent results published by Estrada and coworkers demonstrated that reactive oxygen species (ROS) level increased cellular stress and accumulation of aberrations associated with cellular senescence [26]. Additionally, it was proved that all ex vivo cell expansion procedures favour senescence progression through the accumulation of aneuploid cells [64]. While some authors found that improper expansion could induce premature senescence of MSCs, others demonstrated that MSCs cultured on poly-L-lysine-coated plates could reverse the replicative senescence. Poly-L-lysine surface provides favourable microenvironment where MSCs exhibit higher grow rate and upregulate stemness markers. This method could significantly contribute to the effective preparation of MSCs for cellular therapy [68]. There are reports defining a direct correlation between telomerase (which blocks cell senescence) activity and stem cell function. Hisamatsu and coworkers identified that growth differentiation factor 6 secreted from young MSCs could exert positive effect *in vivo* on aging-associated changes [48]. Age-related changes in MSCs activate senescence-associated secretory phenotype (SASP) and elicit high production of proinflammatory cytokines (IL-1, IL-6, and IL-8) that could abolish the regenerative process [69, 70]. On top of that, in some cases, it has been documented that SASP could promote tumour growth. Senescent MSCs secrete high amount of numerous proteins in comparison to nonsenescent cells. Among factors that reached the highest levels of secretions was LEPTIN, transforming growth factor alpha (TGF*α*), IL-8, interferon gamma (IFN-gamma), VCAM1, interferon beta (IFN-*β*), IL-4, and monocyte chemotactic protein-1 (MCP-1) [71]. There are reports that demonstrated the effect of MSC aging on their migratory ability, differentiation potential, immunomodulation ability, and tumour progression. Recently, Sepúlveda and coworkers showed how the state of cell senescence affects immunoregulatory activity of hMSCs *in vitro* and *in vivo* [72]. Results published by Di and coworkers indicated that senescent UCB-MSC secrete factors that promote migration and proliferation of breast cancer cells. The authors proved that aged MSCs develop characteristic SASP and produced high level of IL-6, which may be a key factor in tumour progression [70].

Multiple advanced methods are now available to establish chromosomal stability of MSCs including FISH (fluorescence in situ hybridization), spectral karyotyping, and comparative genomic hybridization (CGH) [73]. Previous reports demonstrated that conventional karyotyping is not the most effective method for evaluation of genomic stability of MSCs for clinical applications. More attentions should be focused on MSC safety based on traditional Giemsa G-banding combined with FISH [74]. Wang and coworkers compared hMSCs obtained from umbilical cord at senescent stage vs early passage using high-resolution array-based comparative genomic hybridization (aCGH). The group analysed different aspects including telomerase activity and telomere length as well as multipotency of MSCs and their surface markers. The authors also distinguished differences in gene expression profile and genomic stability of investigated cells from an early to a late passage [75]. Thus, the chromosomal stability should be routinely tested in expanded MSCs prior their transplantation *in vivo*.

The issue concerning MSC transformation is controversial. There are reports indicated that MSCs cultured *in vitro* undergo malignant transformation [74, 76, 77]. High rate of aneuploidy was demonstrated in rodent MSCs [78]. In terms of human MSCs, it is not clear whether aneuploidy clones observed *in vitro* represent the senescent or transformed cell population [74]. Moreover, many of publications concerning hMSCs transformation were retracted with regard to possibility of contamination of tumour cell lines due to the use of not reliable verification methods.

The other potential risk of MSC therapy in rodents was reported as possible teratogenic capacity that may not be detected during *in vitro* cell culture but may occur *in vivo* following MSC transplantation [76, 77]. Importantly, such adverse effects as *in vivo* sarcoma formation have been reported for rodent MSCs expanded in prolonged ex vivo culture, which may potentially lead to accumulation of random mutations occurring in highly proliferating cells. Miura and coworkers have shown that murine BM-MSCs, after many passages in culture, gained unlimited cell proliferation potential and underwent malignant transformation resulting in fibrosarcoma formation observed in multiple organs after intravenous administration of cells [76]. Such transformed MSCs generated in long-term culture exhibited accumulated chromosomal abnormalities, increased telomerase activity, and c-myc expression as well as more efficient resistance to anticancer agents [76]. Recently, Jeong and coworkers demonstrated for the first time that malignant transformation of MSCs may happen not only in prolonged culture but also during short-term *in vitro* expansion [77]. The authors provided evidence that murine BM-MSCs harvested at early passages (P3-P6) and injected directly into peri-infarct area of the injured heart or into hind limb muscles of mice with diabetic neuropathy led to tumour formation in 30% and 46% of treated animals, respectively [77]. Genetic analysis of MSC line used in this study revealed accumulative chromosomal abnormalities including fusion, fragmentation, and ring formation. Importantly, these MSCs demonstrated normal features, i.e., morphology or antigenic profile when cultured *in vitro* (at P4) but their abnormal behaviour was proven exclusively after transplantation [77]. Considering the fact that short expansion period (typically 3-5 passages) is a widely used procedure for hMSC propagation prior to transplantation, the fact of possible cell transformation in such early stages of culture should be considered and MSC specimens should be always subjected for detailed karyotype analysis. There are some studies providing the evidence that hMSCs may undergo genetic changes and mutate after prolonged cell culture *in vitro* [79, 80]. Such important findings confirm the commonly known rationale for maximally reduced and limited ex vivo cell manipulation and culture for any cell type including MSCs, especially when the cells are considered as transplant material in humans. Genomic stability plays a critical role in stem cell-based therapies, and thus, it has emerged much attention. Previous reports demonstrated that embryonic stem cells (ESCs) and induced pluripotent stem cells (iPSCs) frequently develop chromosomal abnormalities or DNA copy number variations (CNVs) during cell propagation [81]. However, human MSCs are stem cells in an intermediate state of differentiation between ESCs and adult cells and it is not fully clear whether MSCs can experience adaptive genetic changes during long-term expansion *in vitro*. It needs to be underlined that the most lately appeared studies concerning transformation of MSCs were performed on animal cells, and up to now, abnormalities of human MSCs were not directly proven. Our unpublished results indicate that human MSCs during long-term culture may alter their phenotype into abnormal cells with improper karyotype. Recently, results published by Stultz's group demonstrated that chromosomal aberrations could be clonally propagated throughout long culture-expanded MSCs [82]. Moreover, a variety of studies demonstrated that aneuploidy could be found not only at the late cell passages but also at early passages of *in vitro* culture of human MSCs derived from the bone marrow or adipose tissue [26, 75, 82].

## 5. Labelling Procedure of MSCs May Influence Their Function

An important issue for future clinical applications employing exogenous MSCs is the knowledge of the fate of the transplanted cells in recipient tissues. For this reason, cell labelling facilitates the determination of the degree of engrafted cells in places of expected actions. Proper cell staining enables the study of the migration routes of exogenous cells and their persistence in the recipient organism and allows the visualization of cell differentiation and monitoring the integration process of transplanted cells with the host tissues or the evaluation of elimination process.

Unfortunately, the methods used for exogenous cells labelling are not indifferent to these cells [83]. Obviously, there is a lot of literature reports on the safety of cell labelling methods proving that labelling of cells have no negative impact on their metabolism, the presence of characteristic surface markers, the differentiation potential, or their immunomodulating properties [84–86]. Small particles of iron oxide (SPIO) labelling MSCs might compromise several aspects of cell biology, i.e., migration properties and colony-forming abilities [87]. Functional tests can be used, in which research on the influence of SPIO on MSCs *in vitro* included the use of an external electromagnetic field could be accomplished. Interestingly, the authors stated that in the absence of an external electromagnetic field, the cell biology of the labelled MSCs was not compromised. However, in the presence of the magnetic field, the ability of cell colony formation was significantly affected in SPIO-labelled MSCs. Nevertheless, this effect was transient and after magnetic field cessation, the capacity of cells to form colonies returned to preexperiment values [88]. Besides, the expression of several external membrane markers was changed, as well as differentiation into the adipogenic type was improved. In a different study, iron oxide and protamine sulfate (Fe-Pro) aggregates were reported to cause changes in the expression patterns of several markers of MSCs, i.e., transient increase of MSC marker CD146 and undifferentiating status related gene Oct-4 [89]. In the case of SPIOs, it has been shown that the utilization of this contrast agent could be harmful to MSCs depending on the concentration used. In one study, 25 *μ*g Fe/mL of fluorescent SPIO labelling was found to be safe and did not affect cell proliferation rate and cytoskeletal structures; however, osteogenic differentiation potential was declined [90]. Roeder and coworkers showed that elevated doses of SPIO contribute to the disturbances in chondrogenic MSC differentiation; in particular, the cartilage differentiation was distorted, where specific genes were downregulated along with the increasing dose of SPIO [91]. At highest doses tested, moderate cytotoxicity was demonstrated and no significant negative effects were observed on cell viability or mitochondrial activity. To add with, chondrogenesis was disturbed when MSCs were labelled with fluorescent quantum dots. In this case, chondrocyte specific markers evaluated at mRNA and protein level were significantly downregulated [92]. Besides chondrogenesis, osteogenic differentiation was reported to be affected by SPIO labelling. Gold nanoparticles (Au-NPs) similar to iron containing compounds elicit negative effects on MSCs. Nold and coworkers disclosed that Au-NPs caused concentration-correlated depletion of proliferation and were toxic at high doses [93].

In the field of fluorescent labelling, there are some more examples of the negative influence of this kind of cell tagging on MSC biology. Gallina and coworkers reported that after fluorescent silica nanoparticle (SiO2-NP) MSC labelling, nanoparticles were accumulated in lysosomes and their number was substantially elevated compared to unlabelled cells [94]. Despite high number of lysosomes, neither oxidative stress nor cytotoxicity was noted. Viral transduction with fluorescent protein encoding genes might compromise MSC natural abilities as well. Ji and coworkers reported that MSCs with transduced expression of GFP had lower colony-forming efficiency and unsettled proliferation rate [95]. In other study, in GFP bearing MSCs, the MMP-2 gene expression was increased which suggests the modification of migration ability showed by GFP bearing MSCs [96]. The other types of fluorescent dyes, i.e., fluorescent lipophilic cationic indocarbocyanine dye (DiI) or bromodeoxyuridine (BrdU), apparently lead to the decreased cell viability. The effect of Dil has been found transient but BrdU caused steady decrease of cell viability during the whole cultivation period [97]. On the other hand, fluorescent dye DiD was found to disturb chondrogenesis of DiD-labelled MSCs, where glycosaminoglycan production was heavily diminished [98]. Finally, there are some data reporting that firefly luciferase gene transduction in MSCs caused exclusive diminution in adipogenic differentiation with unaltered chondrogenic and osteogenic differentiation [99].

There is also some evidence that radioactive labelling discomposes MSC proliferation rate. Fludeoxyglucose-18F- (^18^F-FDG-) treated MSCs proliferated slower shortly after ^18^F-FDG administration, followed by an increase in proliferation, to finally return to the proliferation rate same as untreated cells [100].

## 6. Conclusions

In the last few years, considerable progress has been made in terms of MSC transplantation as the therapeutic approach for different diseases. However, one has to be aware that despite of great potential for application in therapeutic approach, MSCs demonstrate a number of limitations. In this review, we intended to emphasize the potential problems and obstacles associated with MSC therapies observed by various investigators including the risk related with manufacturing procedures and with biological properties of the cells. The bothering issue is that the protocols for isolation and expansion of MSCs vary widely between laboratories, which may significantly influence the effectiveness of stem cell therapy. Therefore, it is crucial to develop international standards of MSC manufactures, which should be processed in good medical practice (GMP) grade, cost-effective, clinically practical, and based on facts. Only then, stem cell therapy will become an established and widely adopted treatment. It is also important to define safety of stem cells therapy and determine therapeutic windows in long-term follow-up, as well as monitoring patients for long time in terms of potential risk of tumour transformations. Up to now, it is difficult to conclude about significant beneficial effects of MSC therapy in different diseases due to the still primary stage of research within this field and the variability within the procedures used in different laboratories. Although the results coming from dozens of clinical trials employing MSCs are encouraging, the common clinical use of MSCs is still under development and is not a routine procedure.
